# Incidence of chronic bronchitis in a cohort of pulp mill workers with repeated gassings to sulphur dioxide and other irritant gases

**DOI:** 10.1186/1476-069X-12-113

**Published:** 2013-12-19

**Authors:** Eva Andersson, Nicola Murgia, Tohr Nilsson, Berndt Karlsson, Kjell Torén

**Affiliations:** 1 Department of Occupational and Environmental Medicine, Sahlgrenska University Hospital, Göteborg, Sweden; 2 Section of Occupational Medicine, Respiratory Diseases and Toxicology, University of Perugia, Perugia, Italy; 3 Department of Occupational and Environmental Medicine, Sundsvall Hospital, Sundsvall, Sweden; 4 Department of Public Health and Clinical Medicine, Occupational and Environmental Medicine, Umeå University, Umeå, Sweden; 5 Section of Occupational and Environmental Medicine, Sahlgrenska Academy, University of Gothenburg, Göteborg, Sweden

**Keywords:** (MeSH), Bronchitis, Chronic, Irritants, Sulphur dioxide, Paper

## Abstract

**Background:**

Occupational exposure to irritants is associated with chronic bronchitis. The aim of this study was to elucidate whether repeated peak exposures with respiratory symptoms, gassings, to sulphur dioxide (SO_2_) and other irritant gases could increase the risk of chronic bronchitis.

**Methods:**

The study population comprised 3,060 Swedish pulp mill workers (84% males) from a cohort study, who completed a comprehensive questionnaire with items on chronic bronchitis symptoms, smoking habit, occupational history, and specific exposures, including gassings. 2,037 have worked in sulphite mills. Incidence rates and hazard ratios (HRs) for the observation period, 1970–2000, in relation to exposure and the frequency of repeated gassings to SO_2_ and other irritant gases were calculated.

**Results:**

The incidence rate for chronic bronchitis among workers with repeated gassings was 3.5/1,000 person-years compared with 1.5/1,000 person-years among unexposed workers (HR 2.1, 95% confidence interval (CI) 1.4-3.1). The risk was even higher in the subgroup with frequent gassings (HR 3.2, 95% CI 2.0-5.2), particularly among never-smokers (HR 8.7, 95% CI 3.5-22).

**Conclusions:**

Repeated gassings to irritant gases increased the incidence of chronic bronchitis in our study population during and after work in pulp mills, supporting the hypothesis that occupational exposures to irritants negatively affect the airways. These results underscore the importance of preventive actions in this work environment.

## Background

Textbooks often mention that workers with high exposure to dust and irritants have an increased prevalence of chronic bronchitis [1]. This assumption has recently been confirmed in large, population-based studies, such as the follow-up of the European Community Respiratory Health Survey as well as other European studies, where high exposure to gas and fumes, especially welding fumes, was associated with increased risk for chronic bronchitis, but not with airflow limitation [2-4]. One large study reports no association between exposure to vapours and chronic bronchitis [5]; however, previous data suggest that occupational peak exposure to sulphur dioxide (SO_2_) and chlorine (Cl_2_) compounds may be a risk factor for chronic bronchitis [6].

In the pulp and paper industry, large groups of workers are exposed to a variety of respiratory irritants such as SO_2_ and different bleaching chemicals. In sulphite mills where wood chips are pulped using an acid method, large groups of process workers are frequently subjected to SO_2_ peak exposure [7,8]. In sulphate mills, the wood is pulped using an alkaline method, resulting in a brownish pulp. This pulp is mostly bleached with Cl_2_ compounds, peroxides, or ozone, resulting in peak exposure of the workers to these irritants [9,10].

It is clear from the above that there is a need for expanded knowledge about whether exposure to irritant gases, for example SO_2_, increases the risk of chronic bronchitis. The aim of the present study was to investigate the incidence of chronic bronchitis in association with repeated peak exposures to SO_2_ and other irritant gases, resulting in respiratory symptoms (“gassings”), by comparing exposed and unexposed workers within a cohort of pulp mill workers from four sulphite mills and one sulphate mill.

## Methods

### Cohort

The participants in the current study were selected from a cohort of workers from Swedish sulphite and sulphate mills. The main overall aims were to study respiratory symptoms and irritants, cancer and cardiovascular diseases. Part of the cohort had previously been analysed regarding incidence of asthma and work-related disability respectively [8,11]. The other studies we have done on irritants are from other mills. The cohort members were identified by searching through all personnel files in four Swedish sulphite mills and one sulphate mill. All individuals who were employed there for 6 months or longer at any time between 1940 and 2000 were included, resulting in a cohort of 14,175 workers. For all these individuals, information about the department where employed and employment time was obtained from the personnel files.

For the specific purposes of this study, workers ever employed at the mills between 1 January 1970 and 1 July 2000 and alive in 2000 (n = 7,786) were eligible for the study. Of these, 56 could not be located, 14 died during the survey, and 327 were excluded because they were older than 80 years, leaving 7,389 for the study.

In 2001, a comprehensive questionnaire was mailed to these 7,389 individuals. After two reminders, the response rate was 44% (n = 3,230). The final population (n = 3,060) for this study comprised those workers identified in the personnel files with complete data regarding employment periods and the bronchitis questions, 66.6% of them had been working in sulphite mills. The study was approved by the University of Gothenburg Ethical Committee.

### Definitions

“Chronic bronchitis” was defined as a positive answer to both “Do you have cough with phlegm?” and (if “yes”) “Have you been coughing daily for more than 3 months a year for 2 years?”, according to the American Thoracic Society’s definition of chronic bronchitis [12]. Workers answering “yes” to both questions were also asked to state the year in which the cough with phlegm started. “Asthma” was defined as self-reported physician-diagnosed asthma. “Atopy” was defined as an affirmative answer to questions about allergy in childhood and/or ever having had hay fever. “Smoking” was defined as daily smoking during at least 1 year. Participants were classified, according to their status at follow-up, as current smokers, former smokers or never-smokers. They were also asked about years of starting and stopping smoking as well as the amount smoked per day during each 10-year period, to allow calculation of pack-years. “Exposure to environmental tobacco smoke (ETS) in the workplace” was defined as self-reported exposure to ETS several times a week over the years worked at the pulp mill.

### Exposure

The questionnaire comprised items, used in our previous studies, about occupational history and specific exposures [6,13,14]. Information about the departments in which the subjects had worked, and for how long, was gathered through the mills’ personnel files. Workers were classified as exposed if they reported any occupational exposure to SO_2_ or Cl_2_/chlorine dioxide (ClO_2_) (n = 1,300) or stated that they have had peak exposures giving respiratory symptoms to other irritating chemicals (hydrogen peroxide, hydrogen sulphide, ammonia, diisocyanates, formaldehyde, ozone or others). The exposures were partly overlapping, as shown in Table 1.

**Table 1:** Overlapping exposures for 1,476 exposed pulp mill workers employed at any time between 1970 and 2000

	**Exposed to sulphur dioxide**	**Gassings to sulphur dioxide**	**Exposed to chlorine/chlorine dioxide**	**Gassings to chlorine/chlorine dioxide**	**Gassings to other irritants**
	**(N = 1,009)**	**(N = 624)**	**(N = 868)**	**(N = 494)**	**(N = 632)**
**Exposed to sulphur dioxide**	100%	100%	66.5%	74.1%	58.9%
**Gassings to sulphur dioxide**	61.8%	100%	39.9%	55.5%	46.4%
**Exposed to chlorine/chlorine dioxide**	57.2%	55.5%	100%	100%	52.4%
**Gassings to chlorine/chlorine dioxide**	36.3%	43.9%	56.9%	100%	38.8%
**Gassings to other irritants**	36.9%	47.0%	38.1%	49.6%	100%

**Figure 1:**
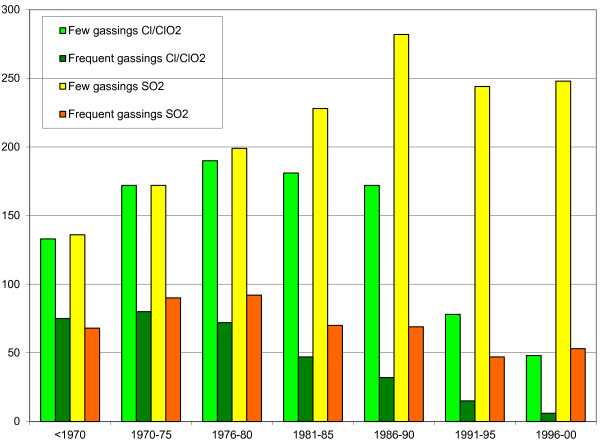
Number of pulp mill workers reporting gassings in different time periods (retrospectively in questionnaires).

The exposed workers (n = 1,476) were further classified on the basis of whether they had experienced gassings. “Gassings” were defined as an affirmative answer to the questions “Have you ever been exposed to “x” resulting in coughing, breathlessness, wheezing or pain in the chest?”, where “x” was SO_2_ or Cl_2_/ClO_2_ or other irritants [6,15]. The workers reporting gassings (n = 1,217) could be further divided by specific answers in the questionnaire into those who had experienced few episodes of gassings (n = 866) and those who had experienced frequent episodes (n = 351). The exposed workers were employed mainly in the pulping, especially digester and bleaching, but also maintenance departments, (Table 2). Gassings due to Cl_2_/ClO_2_ have decreased after 1990 because of change in bleaching practice but not the one due to SO_2_ (Figure 1). Reported exposure to hydrogen peroxide was 22.1%, hydrogen sulphide 25.9%, ammonia 19.3%, diisocyanates 4.3%, formaldehyde 8.5%, ozone 6.9% and others 15,1%. Gassings to any of these other irritants were reported by 20.7% of the workers.

**Table 2:** Distribution of workers in mill departments and exposure frequency in these departments among pulp mill workers in the cohort

**Work departments in pulp mills**	**Unexposed**	**Exposed but no gassings**	**Few episodes of gasssings**	**Frequent gassings**
	**% (N) in department**	**% (N) in department**	**% (N) in department**	**% (N) in department**
All departments, 100% (N = 3060)	51,8% (1,584)	8,5% (259)	28,3% (866)	11,5% (351)
Office ever, 12.8% (N = 391)	73.7% (288)	5.9% (23)	15.9% (62)	4.6% (18)
Office only, 9.4% (N = 289)	86.5% (250)	3.1% (9)	9.0% (26)	1.4% (4)
Wood preparation ever, 10.8% (N = 330)	44.6% (147)	8.8% (29)	31.8% (105)	14.9% (49)
Pulp production ever, 25.6% (N = 783)	21.1% (165)	12.1% (95)	41.8% (327)	25.0% (196)
---Digester ever, 12.2% (N = 372)	17.7% (66)	12.9% (48)	39.3% (146)	30.1% (112)
---Bleachery ever, 6.1% (N = 186)	9.7% (18)	12.9% (24)	46.8% (87)	30.7% (57)
Maintenance ever, 24.5% (N = 749)	45.1% (338)	10.2% (76)	33.4% (250)	11.4% (85)
Paper production ever, 32.6% (N = 997)	56.8% (566)	6.4% (64)	27.3% (272)	9.5% (95)
Paper production only, 17.8% (N544)	75.0% (408)	3.7% (20)	17.8% (97)	3.5% (19)
Not specified, 11.8% (N = 360)	61.4% (221)	6.7% (24)	21.7% (78)	10.3% (37)

66.6% have been working in sulphite mills.

There were 1,584 unexposed workers, who did not report exposure to SO_2_ or Cl_2_/ClO_2_ nor gassings due to the other above mentioned irritating chemicals. The unexposed workers were employed mainly in the paper production departments, in the storage-transportation departments and in office work.

### Statistical methods

The statistical analyses were performed using version 9.1 of the statistical software package SAS (SAS Institute, Cary, NC, USA). Frequencies were compared using a chi-square test and continuous variables were compared using Student’s *t*-test, both with a significance level of p < 0.05. Information about year of onset of chronic bronchitis, years of employment, and episodes of gassings allowed the calculation of incidence of chronic bronchitis after start of employment in relation to any exposure to irritant gases and gassings. The analyses were also performed with a latency period of 5 years, excluding the cases (and person-years) with onset in the first 5 years after start of employment. Person-years were calculated from the start of the first employment period until the year 2000, the age of 70 or (for cases) the year of reported diagnosis, whichever came first; if the subject began to work in the pulp mill before 1970, we used 1970 as the starting point. Hazard ratios (HRs) were calculated from Cox regression models, adjusting for gender, smoking (both current smoking and pack-years), physician-diagnosed asthma, and a time-dependent variable of age (where 0 = <50 years of age and 1 = ≥50 years old). Separate models were performed for different smoking categories. Other models with the same adjustments were calculated considering the departments where the subject had worked as an estimate of the exposure. We also ran the models adjusted for ETS. Stratified analyses for smoking and gassings were performed adjusting for gender, physician-diagnosed asthma, and the time-dependent variable of age.

## Results

Basic data about the study subjects are given in Tables 3 and 4. The examined population comprised 3,060 individuals with a predominance of men (n = 2,558, 84%). A total of 164 participants (5.4%) reported onset of chronic bronchitis between the ages of 16 and 70. However, 17 of these also reported an onset year of chronic bronchitis that occurred before the start of their employment, and ten before 1970 or after 2000, so they were excluded. This resulted in 137 cases (121 men and 16 women) with new chronic bronchitis during or after work in pulp mills during 1970–2000. Most of them (67.9%), contracted chronic bronchitis before the age of 50 years. The prevalence of chronic bronchitis in current smokers was 10.4%, while in former smokers it was 5.6%. Mean number of pack-years among current smokers was 20.4 (SD 12.3) compared with 12.6 (SD 11.7) in former smokers.

**Table 3:** Basic data about the investigated pulp mill workers employed at any time between 1970 and 2000, by exposure to irritant gases

	**All**	**Unexposed**	**Exposed but no gassings**	**Few episodes of gassings**	**Frequent gassings**
	**(N = 3,060)**	**(N = 1,584)**	**(N = 259)**	**(N = 866)**	**(N = 351)**
Mean age at follow-up, yrs (SD)	46.9 (12.4)	45.9 (12.7)	45.5 (11.5)	47.6 (12.3)*	51.2 (11.3)***
Atopy, % (N)	18.4% (564)	17.6% (278)	17.0% (44)	20.2% (175)	19.1% (67)
Physician-diagnosed asthma, % (N)	7.1% (216)	5.4% (85)	4.3% (11)	7.0% (61)	16.8% (59)***
Adult chronic bronchitis, % (N)	5.4% (164)	3.2% (50)	2.7% (7)	6.0% (52)**	15.7% (55)***
Pack-years among current and former smokers, mean (SD)	14.7 (12.4)	14.1 (12.3)	14.1 (12.0)	14.9 (11.8)	17.1 (13.9)**
Current smokers, % (N)	12.9% (395)	13.6% (216)	13.1% (34)	11.8% (102)	12.3% (43)
Former smokers, % (N)	34.8% (1,064)	32.8% (519)	28.2% (73)	37.8% (327)*	41.3% (145)**
Never-smokers, % (N)	51.0% (1,560)	51.8% (820)	57.9% (150)	49.8% (431)	45.3% (159)*
Person-years at risk, sum	57,977	27,802	5,064	17,333	7,778
Person-years at risk, mean (SD)	19.1 (9.1)	17.7 (9.1)	19.6 (8.6)**	20.2 (8.9)***	22.9 (8.4)***

Compared to unexposed *p < 0.05, **p < 0.01, ***p < 0.0001.

*SD* Standard deviation.

**Table 4:** Basic data about the investigated pulp mill workers employed at any time between 1970 and 2000, by smoking status

	**All**^**¤**^	**Never-smokers**	**Ever-smokers**	**Former smokers**	**Current smokers**
	**(N = 3,060)**	**(N = 1,560)**	**(N = 1,459)**	**(N = 1,064)**	**(N = 395)**
Mean age at follow-up, yrs (SD)	46.9 (12.4)	43.5 (12.5)	50.6 (11.2)***	51.5 (11.1)***	48.1 (11.0)***
Atopy, % (N)	18.4% (564)	21.2% (331)	15.4% (224)***	15.1% (161)***	16.0% (63)*
Physician-diagnosed asthma, % (N)	7.1% (216)	7.6% (118)	6.5% (95)	6.4% (68)	6.8% (27)
Adult chronic bronchitis, % (N)	5.4% (164)	4.0% (63)	6.9% (101)**	5.6% (60)	10.4% (41)***
Pack-years among current and former smokers, mean (SD)	NA	NA	14.7 (12.4)	12.6 (11.7)	20.4 (12.3)***
Any exposure to irritant gases, % (N)	48.2% (1,476)	47.4% (740)	49.6% (724)	51.2% (545)	45.3% (179)
Gassings, % (N)	39.8% (1217)	37.8% (590)	42.3% (617)*	44.4% (472)**	36.7% (145)
Frequent gassings, % (N)	11.5% (351)	10.2% (159)	12.9% (188)*	13.6% (145)**	10.9% (43)
Unexposed workers, % (N)	51.8% (1,584)	52.6% (820)	50.4% (735)	48.8% (519)	54.7% (216)
Person-years at risk, sum	57,977	26,548	30,722	22,677	8,045
Person-years at risk, mean (SD)	19.1 (9.1)	17.1 (9.3)	21.3 (8.3)***	21.6 (8.2)***	20.7 (8.8)***

^¤^41 subjects with unknown smoking status could not be classified in smoking categories.

Compared to never-smokers *p < 0.05, **p < 0.01, ***p < 0.0001.

*NA* Not applicable, *SD* Standard deviation.

Ever-smokers were older and reported less atopic conditions then never-smokers (Table 4). Former smokers were more exposed to gassings. The incidence rates and HRs for chronic bronchitis in relation to smoking and exposure to irritants and gassings are shown in Table 5. The incidence for unexposed former smokers was 1.5 per 1.000 person-years and for current smokers 3.7 per 1.000 person-years. There was no decrease in incidence during the years (data not shown). Workers exposed to irritant gases showed an increased risk for onset of chronic bronchitis during their employment, and those workers experiencing gassings had even higher risks, especially those reporting frequent gassings (HR 3.2, 95% CI 2.0–5.2). When the analyses were performed using the 5-year latency model the results were almost identical (data not shown). When in the model, department was used as estimate of the exposure, a higher HR was found in some departments where peak exposure is more likely to occur, such as in the digester area (HR 2.3, 95% CI 1.3–4.2), and in maintenance work (HR 2.0, 95% CI 1.2–3.3). In our study population, working in bleachery carried a risk of chronic bronchitis, though not statistically significant (HR 1.7, 95% CI 0.8–3.5).

**Table 5:** Number of cases (N), incidence rates (IRs) per 1,000 person-years and hazard ratios (HRs)*, with 95% confidence intervals (CI), for chronic bronchitis during and after employment time, 1970–2000, according to reported exposure and gassings to chemical irritants among pulp mill workers, by smoking status

	**All**		**Never-smokers**		**Ever-smokers**	
	**IR (N)**	**HR (95% CI)**	**IR (N)**	**HR (95% CI)**	**IR (N)**	**HR (95% CI)**
Exposed to irritant gases	3.1/1,000 (95)	1.9 (1.3–2.8)	3.1/1,000 (42)	3.0 (1.5–6.1)	3.3/1,000 (53)	1.5 (0.9–2.4)
Any gassings	3.5/1,000 (89)	2.1 (1.4–3.1)	3.5/1,000 (39)	3.7 (1.8–7.7)	3.6/1,000 (50)	1.6 (0.97–2.5)
Few episodes of gassings	2.5/1,000 (43)	1.6 (1.03–2.5)	1.9/1,000 (15)	2.4 (1.03–5.7)	3.0/1,000 (28)	1.3 (0.8–2.3)
Frequent gassings	5.9/1,000 (46)	3.2 (2.0–5.2)	7.5/1,000 (24)	8.7 (3.5–22)	4.9/1,000 (22)	1.9 (1.1–3.5)
Unexposed	1.5/1,000 (42)	1.0	0.9/1,000 (12)	1.0	2.1/1,000 (30)	1.0

*adjusted for gender, physician-diagnosed asthma, both current smoking and pack-years (except models for never-smokers), and time-dependent age ≥50 years.

The risks for former smokers and current smokers were similar for the exposure categories except for frequent gassings where HR for former smokers was 2.1 (95% CI 0.9–4.7) and 1.9 (95% CI 0.7–4.8) for current smokers. Never-smokers with frequent gassings showed a high risk for onset of chronic bronchitis (HR 8.7, 95% CI 3.5–22) (Table 5). Stratified analyses for different categories of smoking and gassings showed a similar risk for current smokers with frequent gassings if compared with unexposed never-smokers (Table 6). When analyses were performed internally among ever-smokers, the chronic bronchitis risk from current smoking and pack-years are similar in unexposed and exposed workers. In a Cox model restricted to exposed ever-smokers, HR for current smoking was 2.2 (95% CI 1.2–4.0), for pack-years 1.02 (95% CI 1.00–1.05) and for frequent gassings 1.5 (95% CI 0.9–2.7).

**Table 6:** Incidence rates per 1,000 person-years and risk of chronic bronchitis, during and after employment time 1970–2000, in different strata of smoking and gassings among pulp mill workers

**Smoking**	**Gassings**	**Cases**	**Incidence**	**Crude risk**	Cox models*
					**HR (95% CI)**
No	No	12	0.9/1,000	1	1
No	Yes	39	3.5/1,000	3.9	3.7 (1.8-7.7)
Ever	No	30	2.1/1,000	2.3	2.5 (1.2-5.0)
Ever	Yes	50	3.6/1,000	4.0	3.9 (1.9-7.8)
**Current smoking**	**Frequent gassings**	**Cases**	**Incidence**	**Crude risk**	Cox models*
					**HR (95% CI)**
No	No	12	0.9/1,000	1	1
No	Yes	24	7.5/1,000	8.3	8.7 (3.5-22)
Yes	No	15	3.7/1,000	4.1	3.7 (1.6-8.1)
Yes	Yes	7	7.4/1,000	8.2	10.6 (3.1-36)

Crude risk and hazard ratios (HRs)* with 95% confidence intervals (CI), for chronic bronchitis.

*adjusted for gender, physician-diagnosed asthma and time-dependent age ≥50 years.

Asthma is associated with chronic bronchitis, 18% of the asthmatics had also chronic bronchitis, therefore we also ran the analyses excluding all subjects with physician-diagnosed asthma (n = 207). Any gassings (HR 1.8, 95% CI 1.1–2.8) and frequent gassings (HR 2.5, 95% CI 1.4–4.5) were still associated with an increased risk for onset of chronic bronchitis. Similar findings were also found among never-smokers and ever-smokers (data not shown). We finally ran the analysis also including exposure to ETS as a confounder but the results did not change (data not shown).

## Discussion

### Main findings

The results of this study, based on a retrospective analysis of data on a cohort of pulp mill workers, support the hypothesis that exposures to irritant gases could contribute to induce chronic bronchitis. Furthermore, the risk for onset of chronic bronchitis seems higher in workers experiencing peak exposures to irritants giving respiratory symptoms, here named “gassings”.

Even if the incidence rate of chronic bronchitis was lower among never-smokers, the risk for new-onset chronic bronchitis related to irritants was higher in this group compared with smokers, suggesting a strong role for occupational exposure in the aetiology of chronic bronchitis in non-smokers.

Unexposed participants showed an incidence rate of 1.5 per 1,000 person-years for chronic bronchitis, which breaks down to 2.1/1,000 person-years for ever-smokers and 0.9/1,000 person-years for never-smokers. These results compare well with data from a general population study where the incidence rate of chronic bronchitis was 1.9/1,000 person-years, breaking down to 2.4/1,000 person-years for ever-smokers and 1.3/1,000 person-years for never-smokers [4]. These figures are quite close to our estimates, supporting the validity of our findings.

Compared to unexposed never-smokers both ever-smokers and never-smokers have the same risk when exposed to gassings, but the exposed ever-smokers do not have an additive risk for smoking. Our results, with a lack of additive effect of occupational exposure and smoking, are unexpected; as a matter of fact, in other studies the exposure to vapours, gas, dust and fumes was reported to have at least an additive effect with tobacco smoke regarding the risk of chronic bronchitis [16,17]. However, it is important to point out that the studies reporting this additive effect were performed in the general population with lower exposure to irritants even if also exposed to other risk factors, while we have assessed a cohort of pulp mill workers considering a very high exposure to irritants. Being aware of the healthy worker effect that is usually seen in cohort studies on chronic bronchitis [18], healthy subjects, irrespective of exposure to irritants and gassings, may continue smoking. Indeed, in our population, reported pack-years for workers who experienced frequent episodes of gassings were greater compared with those for unexposed workers or workers with a few gassings. Anyway former smokers had higher exposure to gassings and there are more former smokers among the workers reporting gassings than among unexposed. This latter result could be explained by the fact that some smoker had probably stopped smoking due to the gassings. In our study the total dust exposure in the last 40 years was lower [19] compared with that reported in other studies involving paper dust and chronic bronchitis [20]. Irritants other than those defined in this study have been considered previously [19], but since their airborne concentration was fairly low, they were judged insignificant in this study. To support the hypothesis that other exposures were irrelevant in our cohort there is the finding that the incidence ratio of the disease in not exposed ever-smokers and never-smokers is lower than in general population-based European studies [4].

### Validity issues

One major limitation of this design is recall bias, particularly exposure dependent recall bias. Recall of chronic bronchitis may be higher among people who are exposed than among their unexposed counterparts, introducing falsely increased risk estimates. In a similar way, self-reported occupational exposure data could be differentially misclassified by disease status, and appear higher among those with chronic bronchitis [21]. However, prompted questions about exposure, as used in this study, are less likely than open-ended questions to be subject to recall bias [22]. The questionnaire study in the cohort was conducted mainly for respiratory symptoms and irritants but in the questionnaire there was a lot of other topics and the given aim was to study work environment and health.

The reported year of onset for chronic bronchitis may be sensitive to misclassification, meaning that a subject may report an incorrect year. We are not aware of any studies validating the self-reported year of diagnosis or disease onset among people with chronic bronchitis. However, in another study, reported year of asthma onset has been shown to be fairly accurate, deviating from reality by only a few years; and the deviation was not associated with atopy, smoking or gender [23].

The response rate in the present study was 44%, which may have introduced bias. In this cohort, we have previously analysed a random sample of non-responders regarding the question of physician-diagnosed asthma and gassings, and found no difference regarding the risk of asthma between responders and non-responders [8]. There was no difference in exposure to gassings or frequent gassings, ever-smokers or current smokers, or asthma among responders and 254 telephone interviewed non-responders in the present enlarged cohort but chronic bronchitis was not asked for. The non-responders were 2.5 years younger though. In another study, when adjusted for confounders and exposures, the prevalence of respiratory symptoms did not vary significantly between responders and non responders followed up by telephone [24,25]. Hence, at least regarding respiratory outcomes and occupational exposures, there are some studies indicating that the exposure-response relationships seem to be only marginally affected by non-response bias.

## Conclusions

The associations observed in the present retrospective study should ideally be further investigated in a prospective study including recurring health examinations and exposure assessments. However, there are great problems with capturing peak exposure to irritants by means of continuous personal monitoring. For this reason, additional methods such as diary entries and area samplings must be considered.

In conclusion, repeated peak exposures to irritant gases giving rise to respiratory symptoms increased the incidence of chronic bronchitis among pulp mill workers, supporting the hypothesis that occupational exposure to irritants negatively affects the respiratory tract. These results underscore the importance of preventive actions in this work environment.

## Abbreviations

Cl2: Chlorine; ClO2: Chlorine dioxide; CI: Confidence intervals; ETS: Environmental tobacco smoke; HR: Hazard ratios; IR: Incidence rate; N: Number; SD: Standard deviation; SO2: Sulphur dioxide.

## Competing interests

The authors declare that they have no competing interests.

## Authors’ contributions

KT, EA and TN designed the study. EA, BK and KT were responsible for data collection in the mills. EA, NM and KT managed and analysed the data. All authors participated in the interpretation and final drafting of the manuscript. All authors read and approved the final manuscript.
